# High phenylalanine concentrations induce demyelination and microglial activation in mouse cerebellar organotypic slices

**DOI:** 10.3389/fnins.2022.926023

**Published:** 2022-09-29

**Authors:** Orli Thau-Zuchman, Patrick N. Pallier, Paul J. M. Savelkoul, Almar A. M. Kuipers, J. Martin Verkuyl, Adina T. Michael-Titus

**Affiliations:** ^1^Centre for Neuroscience, Surgery and Trauma, Barts and The London School of Medicine and Dentistry, The Blizard Institute, Queen Mary University of London, London, United Kingdom; ^2^Danone Nutricia Research, Utrecht, Netherlands

**Keywords:** white matter, phenylketonuria, organotypic slices, phenylalanine, microglia, cerebellum

## Abstract

Phenylketonuria (PKU) is an inborn error of metabolism. Mutations in the enzyme phenylalanine hydroxylase (PAH)-encoding gene lead to a decreased metabolism of the amino acid phenylalanine (Phe). The deficiency in PAH increases Phe levels in blood and brain. Accumulation of Phe can lead to delayed development, psychiatric problems and cognitive impairment. White matter (WM) damage is a neuropathological hallmark of PKU and can be seen even in early detected and treated PKU patients. The mechanisms linking high Phe concentrations to WM abnormalities remain unclear. We tested the effects of high Phe concentrations on myelin in three *in vitro* models of increasing complexity: two simple cell culture models and one model that preserves local brain tissue architecture, a cerebellar organotypic slice culture prepared from postnatal day (P) 8 CD-1 mice. Various Phe concentrations (0.1–10 mM) and durations of exposure were tested. We found no toxic effect of high Phe in the cell culture models. On the contrary, the treatment promoted the maturation of oligodendrocytes, particularly at the highest, non-physiological Phe concentrations. Exposure of cerebellar organotypic slices to 2.4 mM Phe for 21 days *in vitro* (DIV), but not 7 or 10 DIV, resulted in a significant decrease in myelin basic protein (MBP), calbindin-stained neurites, and neurites co-stained with MBP. Following exposure to a toxic concentration of Phe, a switch to the control medium for 7 days did not lead to remyelination, while very active remyelination was seen in slices following demyelination with lysolecithin. An enhanced number of microglia, displaying an activated type morphology, was seen after exposure of the slices to 2.4 mM Phe for 10 or 21 DIV. The results suggest that prolonged exposure to high Phe concentrations can induce microglial activation preceding significant disruption of myelin.

## Introduction

Phenylketonuria (PKU; OMIM # 261600) is an inherited autosomal recessive metabolic disorder that affects phenylalanine (Phe) metabolism. It is caused by a deficiency in the enzyme phenylalanine hydroxylase (PAH; EC 1.14.16.1) (Konecki et al., 1992; Williams et al., 2008). Wild-type PAH converts Phe into tyrosine, requiring the cofactor tetrahydrobiopterin (BH4), iron and oxygen (Blau et al., 2010). PAH gene mutations alter its activity and promote protein misfolding. These changes trigger a cellular stress response and degradation of the abnormal proteins. The steady-state level of the mutant enzyme is reduced and there is an accumulation of Phe in blood and brain (Waters, 2003). Based on plasma Phe concentrations, PAH deficiency can be classified into mild/atypical PKU (Phe at 0.6–0.9 mM), moderate PKU (Phe at 0.9–1.2 mM), and classic PKU (Phe > 1.2 mM) (Preissler et al., 2016), compared to normal levels of about 0.12 mM.

If left untreated, elevated Phe levels can result in a variety of deficits: cognitive impairment, motor impairment, seizures, sleep disturbances, behavioral and psychiatric symptoms (Bilder et al., 2017; van Wegberg et al., 2017). A Phe-restricted diet (Bickel et al., 1953) can be introduced early for management of the condition and prevention or amelioration of neurocognitive impairments (MacCready, 1974). However, in many adults, sub-optimal treatment and lack of adherence to the diet lead to blood Phe levels that exceed the recommended range, with very unfavorable outcomes (Bilder et al., 2017). It is estimated that the number of adults strictly remaining on diet is less than 50% (Guest et al., 2013).

White matter (WM) damage is a neuropathological hallmark of PKU and its deleterious impact on the central nervous system (CNS). In untreated patients, diffuse WM pathology is more likely to reflect hypomyelination, while myelination would proceed close to normal in early treated PKU patients, where the lesions are more likely to reflect demyelination or dysmyelination (Anderson and Leuzzi, 2010). The amount and extent of WM lesions correlate with plasma Phe concentration (Dyer, 1999; Williams et al., 2008) but not brain Phe concentration (Leuzzi et al., 2000). In late-detected subjects, brain Phe concentration correlates with the clinical phenotype better than plasma Phe (Leuzzi et al., 2000). The impaired myelination could be linked to a toxic effect of high brain Phe levels on oligodendrocytes (OLs) (Dyer et al., 1996; Anderson and Leuzzi, 2010), whose sensitivity to elevated Phe levels varies with the CNS developmental stage (Levy et al., 1996; Leuzzi et al., 2000).

Myelination involves the differentiation of OLs into mature cells that express genes for myelin components, e.g., myelin basic protein (MBP) (Baumann and Pham-Dinh, 2001; Jackman et al., 2009). In the human brain, myelination begins in the second trimester of pregnancy within the brainstem and progresses to the cerebellum and the cerebrum, with a period of rapid myelination of virtually all tracts within the first 2 years of postnatal life; further myelination continues into the third decade of life, parallel with the time course of maturation of cognitive function (Aubert-Broche et al., 2008; Shoykhet and Clark, 2011; Kinney and Volpe, 2018). In the cerebellum and brain hemispheres, the median age at which mature myelin is observed is 17.5–27 months (Kinney and Volpe, 2018). OLs that are sensitive to elevated Phe levels would be located mainly in those WM tracts that myelinate after birth, within the forebrain and cerebellum, whereas Phe-insensitive OLs would reside mainly in tracts that myelinate before birth, within the hindbrain and spinal cord (Dyer et al., 1996; Dyer, 1999). There is also evidence of grey matter (GM) abnormalities in PKU (Christ et al., 2016), and since axons and their myelin sheath form a functional unit, loss of myelin could lead to axonal instability (Dyer, 1999).

There remain many gaps in our understanding of the pathophysiology underlying the brain dysfunction reported in PKU patients, which still occurs despite the early detection and management of patients with a low Phe diet. Early diagnosed and well-managed patients may still experience deficits in executive function, attention and processing speed and some psychosocial difficulties, which may influence their quality of life (Enns et al., 2010; Gentile et al., 2010; Bilder et al., 2017). Such deficits might be linked to microstructural WM abnormalities, as supported by imaging studies (White et al., 2010; Antenor-Dorsey et al., 2013; Wesonga et al., 2016; Gonzalez et al., 2018). Despite attempts to characterize the effects of high Phe concentrations on neurons and non-neuronal cells *in vitro* and *in vivo* (Dyer et al., 1996; Dyer, 2000; Hörster et al., 2006; Preissler et al., 2016), the deleterious effect of Phe on OLs and myelination is still not understood. A study that tested high concentrations of Phe (1.6 mM and 5 mM) and its metabolites in rat oligodendrocyte (OL)-enriched cultures or mouse neuronal-glial myelinating cultures, failed to detect any toxic effects on OLs or myelination (Schoemans et al., 2010).

We chose to use a series of three *in vitro* models of increasing complexity (a rat glial precursor cell culture; an OL-enriched culture; a cerebellar organotypic slice culture –OSC) to test the effects of high Phe concentrations on myelination. The most complex of these models, the cerebellar OSC, was selected because the neural circuits, synaptic organization, and cell-cell and cell-matrix interactions that exist in brain tissue are largely preserved in this model. In rodents, myelination is very active 2 weeks after birth, with a peak at around P20 (Semple et al., 2013). The myelination of Purkinje cell axons in cerebellar cultures follows a temporal pattern similar to that seen *in vivo* (Doussau et al., 2017); most myelination occurs postnatally and can be monitored *in vitro* (Notterpek et al., 1993). In rats, cerebellar OSCs are considered comparable to “mature” cerebellum when they reach the equivalent of P15 age (P12 in mouse) (Clancy et al., 2001), where most Purkinje cell axons are myelinated (Dusart et al., 1997). In mice, all of the cerebellum but the most rostral lobules is myelinated by P12, and myelination appears complete by P23 (Foran and Peterson, 1992). In cerebellar OSCs, myelinated axons degenerate after a few days *in vitro* (Dusart et al., 1997; Doussau et al., 2017) but this does not apply to Purkinje cell axons (Dusart et al., 1997). This allows quantifying myelination on a uniform population of axons, compared to other brain slice cultures where myelinated axons of different diameters are present. The WM status of the OSCs can be assessed using MBP immunoreactivity as a marker of OL maturation and myelin. Neurites can be visualized with calbindin-D 28K (calbindin) immunostaining. Calbindin is a calcium-binding protein expressed at a high level in the cerebellum, in particular in Purkinje cell somas, dendrites and axons, and to a lesser extent in spinocerebellar and olivocerebellar afferents (Scotti, 1995). Because microglia play a critical role in the clearance of myelin debris following injury (Hanisch and Kettenmann, 2007; Neumann et al., 2009), we also explored the response of microglia to Phe exposure in the cerebellar OSCs. As in other *in vitro* studies (Schoemans et al., 2010; Schlegel et al., 2016), the range of Phe concentrations we tested reflected the increased plasma Phe levels seen in PKU. In the OSC model, we used an outbred mouse strain to reflect real-life genotype diversity and avoid inbred strain-related potential biases.

## Materials and methods

### Cell cultures

#### Rat glial precursor cell cultures

Rat glial precursor cells (RGPCs) (Invitrogen, Paisley, UK; cat. # N7746-100) were handled according to the supplier’s instructions. After thawing, the cells were cultured in a Cellstart-coated (Thermo Fisher Scientific, UK; cat. # A1014201) T25 flask in complete growth medium containing KnockOut Dulbecco’s Modified Eagle’s Medium (DMEM)/F12 (Thermo Fisher Scientific, UK; cat. # 12660-012) with StemPro NSC SFM supplement (Thermo Fisher Scientific, UK; cat. # A10508-01), EGF (Thermo Fisher Scientific, UK; cat. # PHG0314), basic fibroblast growth factor (Thermo Fisher Scientific, cat. # PHG0024), Glutamax™ I (100X) supplement (Gibco™, Thermo Fisher Scientific, UK; cat. # 35050-038), Platelet-Derived Growth Factor-AA (PDGF-AA) (Thermo Fisher Scientific, UK; cat. # PHG0035) and penicillin/streptomycin (Thermo Fisher Scientific, UK; cat. # 15140148) at 37°C and 5% CO_2_. The next day, the medium was replaced with a fresh growth medium. The old growth medium was centrifuged at 300 g for 7 min and the resuspended pellet was returned to the T25 flask. After 2 days of culture, the cells were removed from the flask with Accutase (Thermo Fisher Scientific, UK; cat. # A11105-01) and they were either used for plating or transferred to a T75 flask. Plated cells were seeded into poly-D-lysine-coated (Sigma-Aldrich, UK; cat. # P6407) black 96-well plates with a clear bottom (Costar, UK; cat. # 3603) at a density of 5,000 cells/well in 100 μl of growth medium. After 24 h, the growth medium was replaced with a differentiation medium containing KnockOut DMEM/F12 with StemPro NSC SFM supplement, 2% fetal bovine serum (FBS) (Thermo Fisher Scientific, UK; cat. # 16141061), Glutamax™ supplement, and penicillin/streptomycin with or without additional L-Phe (Sigma-Aldrich, UK; cat. # P2126), and cells were cultured for an additional 3 or 6 days.

#### Oligodendrocyte-enriched cultures

The protocol was adapted from O’Meara et al. (2011). Cortical cells were extracted from post-natal day 2 (P2) C57BL/6 mouse pups. Oligodendrocyte precursor cells were grown for 10 days at 37°C and 5% CO_2_ in a mixed glial cell culture medium (MGCM) made of DMEM (Sigma-Aldrich, UK; cat. # D6429), 10% FBS (Gibco™, Fisher Scientific, UK; cat. # 16000036), 0.33% penicillin-streptomycin (Merck Millipore, Hertfordshire, UK; cat. # 15140122), and 1% Glutamax™ I (100x) supplement (Gibco™, Thermo Fisher Scientific, UK; cat. # 35050-038), in T25 flasks coated with 1 mg/ml poly-L-lysine (Sigma-Aldrich, UK; cat. # P2636). On post-seeding day one, a full medium change was performed to promote culture viability by removing debris caused by the cell trituration and the death of non-glial cells. Two-thirds of the MGCM medium were refreshed on post-seeding days 3 and 6; on day 6, the MGCM was supplemented with 5 μg/ml of insulin (Sigma-Aldrich, UK; cat. # I6634) (MGCMIns). On day 9, an OL-enriched cell suspension was obtained by shaking the flasks gently (50 rpm) for 45 min to detach microglial cells. The medium was then replaced with 5 ml of fresh MGCMIns and, 3 h later, the flasks were shaken vigorously (220 rpm) for 23 h to detach and collect the OL precursor cells (OPCs) from the bed of astrocytes onto which they had been growing and multiplying. The resulting OPC-enriched MGCM was collected into two 10-cm tissue culture dishes and transferred to the CO_2_ incubator for 15 min for further OPC enrichment of the culture (remaining microglial cells dissociate from the OPCs by gravity and attach strongly to the plastic of the dishes). The resulting OPC-enriched cell suspension was centrifuged and then resuspended in a medium warmed at 37°C made of DMEM containing high glucose (4,500 mg/l) (Sigma-Aldrich, UK; cat. # 6429), 2% B-27™ supplement (Thermo Fisher Scientific, UK; cat. # 11530536), 100 μg/ml of bovine serum albumin (BSA) (Sigma-Aldrich, UK; cat. # A4503), 50 ng/ml of ciliary neurotrophic factor (PeproTech EC Ltd., UK; cat. # 450-50-25), 0.5% FBS (Thermo Fisher Scientific, UK; cat. # 16000044), 1% Glutamax™ I (100x) supplement (Gibco™, Thermo Fisher Scientific, UK; cat. # 35050-038), 49.5 μg/ml of holo-transferrin (Sigma-Aldrich, UK; cat. # T0665), 5 μg/ml insulin (Sigma-Aldrich, UK; cat. # I6634), 60 ng/ml of progesterone (Sigma-Aldrich, UK; cat. # P8783), 16 μg/ml of putrescine (Sigma-Aldrich, UK; cat. # P7505), 5 ng/ml of sodium selenite (Sigma-Aldrich, UK; cat. # S5261), and 400 ng/ml of 3,3′,5-triiodo-L-thyronine (Sigma-Aldrich, UK; cat. # T0281). This medium is known to promote OL differentiation and survival. The cell suspension was then filtered through a 40-μm cell strainer to remove cell clumps, and the cell density was estimated using a Trypan blue exclusion test of cell viability. The OPCs were plated in 1 ml of OL medium with or without additional L-Phe (Sigma-Aldrich, UK; cat. # 91331), at 12,500 cells/cm^2^, onto 0.1 mg/ml poly-D-lysine-coated round cover glasses (VWR, UK; cat. # 631-1578) in 24-well plates, and the culture was maintained by regular two third changes of the OL medium.

### Organotypic slice cultures

#### Origin of materials

The cultures were produced using tissue from CD-1 mouse pups. Dams (CD-1^®^ International Genetic Standard mice, strain code: 022) were obtained from the breeder (Charles River, UK), and maintained and humanely culled in accord with the UK Animals (Scientific Procedures) Act 1986 Amendment Regulations 2012 (SI 2012/3039) and the EU Directive 2010/63/EU. The day of birth was P0. A total of 48 pups were used. The pups were selected at random and were not differentiated according to gender.

**FIGURE 1:**
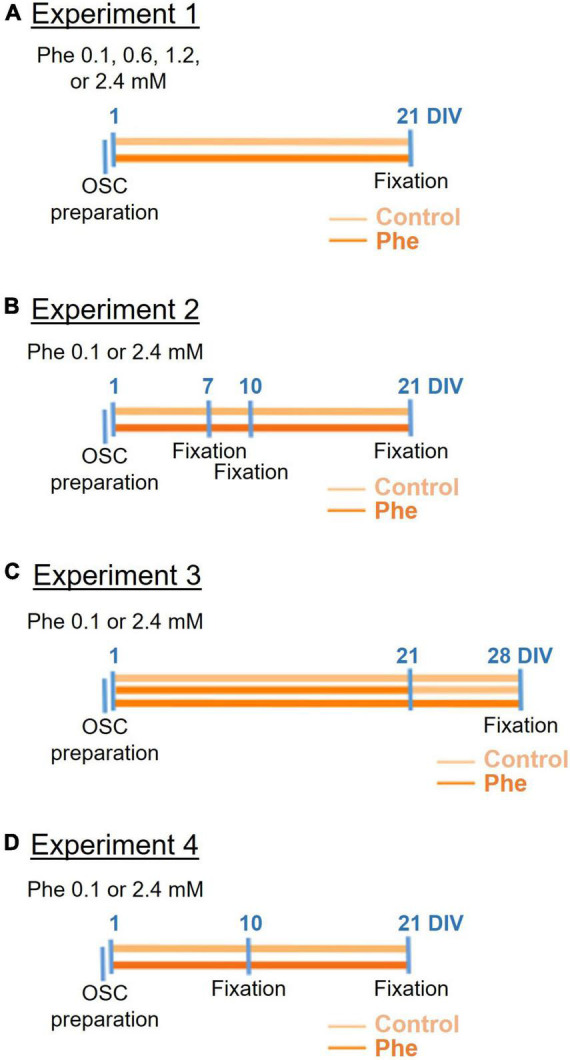
Timeline of the four cerebellar organotypic slice culture experiments. Following preparation of the cerebellar organotypic slices (OSCs) (first blue line on the left), phenylalanine (Phe) was applied to the slices at 1 day *in vitro* (DIV) either at 0.1, 0.6, 1.2, or 2.4 mM (in experiment 1, **A**) or 0.1 or 2.4 mM (in all following experiments, **B–D**) and then the slices were fixed at different time points depending on the experiment. In all experiments, half of the medium was replaced 24 h after the OSC preparation, and then every 3 days (not shown on the timelines). In experiment 3 **(C)**, Phe was replaced by the control medium at 21 DIV in half the slices treated with Phe.

This exploratory study was not pre-registered. All materials and reagents were bought and renewed at the time the experiments were conducted, between 2016 and 2020. The timeline of experiments is summarized in Figure 1.

#### Culture preparation

The cerebellar OSC preparation was adapted from published protocols (Stoppini et al., 1991; Dusart et al., 1997). Cerebellar parasagittal slices were collected from pups at P8, a stage at which a good survival of slices is expected (Dusart et al., 1997). The pups were decapitated, then each brain was dissected out and the cerebellum was isolated and kept in ice-cold sterile, low-sodium artificial cerebrospinal fluid (ACSF) containing 124 mM NaCl, 3 mM KCl, 1.25 mM KH_2_PO_4_, 4 mM MgSO_4_, 2 mM CaCl_2_, 26 mM NaHCO_3_, 10 mM D-(+)- glucose, 2 mM ascorbic acid and 0.075 mM adenosine, at pH 7.3. The ACSF was bubbled with Carbogen (95% oxygen and 5% carbon dioxide) before use. Four hundred μm thick slices were cut using a McIlwain tissue chopper (CLE, UK) and gently separated. Four slices were placed onto the upper chamber of each 0.4-μm pore cell culture insert (Merck Millipore, Hertfordshire, UK; cat. # PICM03050) of a pre-incubated 6-well plate (Sigma-Aldrich, UK; cat. # CLS3506), along with 1 ml of sterile medium containing 50% Minimum Essential Medium Eagle (MEM; Invitrogen, Paisley, UK; cat. # 1109008), 25% heat-inactivated horse serum (Thermo Fisher Scientific, MA, USA; cat. # 16050122), Hanks’ Balanced Salt Solution (HBSS; Thermo Fisher Scientific, MA, USA; cat. # 11530476), 25 mM HEPES buffer solution (Invitrogen, Paisley, UK; cat. # 15630), 1 mM L-glutamine (Invitrogen, Paisley, UK; cat. # 25030), 1 mg/L of insulin (Sigma-Aldrich, UK; cat. # I6634), 0.4 mM L-ascorbic acid (Sigma-Aldrich, UK; cat. # A0278) and s00 U/mL of streptomycin (Invitrogen, Paisley, UK; cat. # 15140). The plate was placed in an incubator at 37°C and 5% CO_2_. In all experiments, half of the medium was replaced 24 h after the slice preparation, and then every 3 days, with a fresh medium enriched in Phe (treated wells) or a non-enriched fresh medium (control wells).

### Phenylalanine application

Wells or slices were arbitrarily allocated to the different treatment groups and/or testing conditions. In all instances, Phe concentrations were chosen to reflect blood concentrations seen in PKU patients. Phe levels exceeding 1.2 mM are considered typical of classic PKU, rising to 2.4 mM in some cases (Dyer, 1999), while levels around 0.6–0.9 mM usually represent mild cases (Williams et al., 2008). Depending on the *in vitro* model, Phe concentration in the control medium was 0.1 mM (OSCs), 0.2 mM (RGPC cultures), or 0.4 mM (OL-enriched cultures), coming from the MEM, KnockOut DMEM/F12, or DMEM media, respectively, which are either equivalent to levels found in the normal human population (<0.12 mM) (Dyer, 1999) or correspond to levels below those seen in mild PKU (0.6–0.9 mM).

#### Rat glial precursor cell cultures

Twenty-four hours post-plating, RGPCs were submitted to either 0.2 (control wells), 1, 5, or 10 mM Phe for either 3 or 6 days –the medium was not refreshed over this period. Three replicates of each dose were made for each culture, and the experiment was replicated over three different cultures.

#### Oligodendrocyte-enriched cultures

On the day of plating onto cover glasses, OPCs were submitted to either 0.4 (control wells), 1.2, 2.4, or 4.8 mM Phe for 14 days. Two-thirds of the OL control medium or OL medium supplemented with L-Phe, as appropriate to each well, were refreshed on post-plating days 6, 10, and 12. Three replicates of each dose were made over two cultures.

#### Organotypic slice cultures, experiment 1: Testing the effect of increasing phenylalanine concentrations on myelin

We tested three different concentrations of Phe by applying to the slices for 21 days *in vitro* (DIV) either a medium enriched with Phe to a final concentration of 0.6, 1.2, or 2.4 mM or a control medium non-enriched in Phe (0.1 mM Phe) (Figure 1A).

#### Organotypic slice cultures, experiment 2: Testing different exposure times of high phenylalanine on myelin

Following the dose-effect exploration, studies continued with the highest Phe concentration of 2.4 mM. First, we incubated slices over different periods (7, 10, or 21 DIV) with either a Phe-containing medium or the control medium (Figure 1B). Then, in a separate experiment, we confirmed that exposing the cerebellar slices to 2.4 mM Phe for 21 DIV led to significant demyelination.

#### Organotypic slice cultures, experiment 3: Testing the effect of stopping high phenylalanine on remyelination

In this experiment, we tested whether spontaneous remyelination would occur after the toxic Phe concentration was reduced back to control levels. We treated slices with 2.4 mM Phe for 21 days, then replaced the Phe-enriched medium with the control medium and maintained the slices for a further 7 days under this condition, after which they were fixed, at 28 DIV. We used a separate control group where slices were exposed to Phe continuously for 28 days until fixation (Figure 1C).

#### Organotypic slice cultures, experiment 4: Testing the effect of high phenylalanine on microglial activation

For this experiment, cerebellar slices were incubated either with a medium containing 2.4 mM Phe or the control medium, for either 10 or 21 DIV (Figure 1D).

### Lysolecithin demyelination/remyelination control experiment

Organotypic slice cultures were exposed to a medium supplemented with the bioactive lipid lysolecithin (lysophosphatidylcholine) to induce demyelination, as has been reported previously by Birgbauer et al. (2004). At DIV7, the culture medium was removed and replaced with a medium supplemented with 0.5 mg/ml of lysolecithin (Cambridge Bioscience, UK; cat. # 24331-10mg-CAY) for 15 to 17 h (Supplementary Figure 4). Then the toxic culture medium was switched back to the control medium and the culture continued for either another 2 days (DIV 10) or 13 days (DIV 21), at which point the slices were fixed and processed for immunostaining.

### Immunostaining

#### Rat glial precursor cell cultures

The cells were fixed at 4 or 7 DIV for 15 min, at room temperature, by directly adding 8% paraformaldehyde to the wells to a final 4% solution in the culture medium. The fixative was then removed and the cells were washed once with 0.01 M phosphate-buffered saline (PBS) and stored at 4°C in PBS containing 0.1% Na azide until staining. When ready for staining, PBS was removed from the wells and the cells were blocked with 5% normal goat serum and 0.1% Triton-X100 in PBS for 1 h at room temperature. To identify differentiated RGPCs, the cells were incubated overnight at 4°C with a mouse anti-galactocerebroside (GalC) antibody (Merck Millipore, Hertfordshire, UK; cat. # MAB342, RRID:AB_94857) diluted 1:500 in the blocking buffer. The cells were then washed three times with PBS and were incubated for 1 h at room temperature with a CF™488A goat anti-mouse antibody (1:1,000; Sigma-Aldrich, UK; cat. # SAB4600388). The cells were then washed three times before Hoechst 33342 (Fisher Scientific, UK; cat. # 10150888) was added to the wells to a final concentration of 2 μg/ml for nucleus staining. On completion, the plates were sealed with Parafilm^®^ and kept in the fridge until imaging.

#### Oligodendrocyte-enriched cultures

The cells were fixed at 14 DIV and immunostained using a variation of the fixation and staining protocols described above (5 min in 8% paraformaldehyde in an equivalent volume of culture medium followed by 5 min in 4% paraformaldehyde and 3 washes in PBS, for fixation; a 10% normal goat serum and 0.1% Triton-X100 in PBS was used for blocking and Hoechst 33342 was used at 1 μg/ml, for immunostaining). The cover glasses were incubated overnight at room temperature with a mouse antibody against MBP (1:100; Abcam, Cambridge, UK; cat. # ab62631, RRID:AB_956157) followed by a goat anti-mouse IgG Alexa Fluor™ 488 (Thermo Fisher Scientific, MA, USA; cat. # A-11001, RRID:AB_2534069). On completion, the cover glasses were mounted onto histology slides using DAPI Fluoromount-G^®^ (SouthernBiotech, Cambridge Bioscience, UK; cat. # 0100-20), sealed in place with nail varnish, and kept in the fridge until imaging.

#### Organotypic slice cultures

At either 7 (data not shown), 10, 21, or 28 DIV, slices were fixed with 4% paraformaldehyde for 40 min at room temperature, followed by three washes, 10 min each, with PBS.

The immunostaining protocol was adapted from Birgbauer et al. (2004). The slices were washed in cold PBS for 15 min three times, then a permeabilization solution (2% Triton X-100 in PBS) was added for overnight incubation at 4°C, followed by incubation with a blocking solution (20% BSA with 0.2 Triton X-100 in PBS) overnight at 4°C. Myelin and axons were detected using antibodies against MBP (1:100; rat anti-MBP, Bio-Rad Laboratories, CA, USA; cat. # MCA409S, RRID:AB_325004) and calbindin (1:1,000; rabbit anti-calbindin, Abcam, Cambridge, UK; cat. # ab11426, RRID:AB_298031), respectively. Co-staining of calbindin and MBP was used to assess the percentage of neurites covered by myelin. Microglia/macrophages were stained separately with an Iba1 antibody (1:600; rabbit anti-Iba1, Wako, Germany; cat.# 019-19741, RRID:AB_839504). All primary and secondary antibodies were diluted in 5% BSA in PBS. Each slice was cut around, separated from the framed insert and placed in a 24-well plate (Starlab, UK; cat. # CC7672-7524) for overnight incubation with the primary antibodies at 4°C. On the following day, the slices were washed three times with 5% BSA in PBS for 30 min to 1 h at room temperature. The secondary antibodies, donkey anti-rat IgG Alexa Fluor™ Plus 488, donkey anti-rabbit IgG Alexa Fluor™ Plus 555, and donkey anti-rabbit Alexa Fluor™ Plus 488 (1:200; Thermo Fisher Scientific, MA, USA; cat. # A48269, RRID:AB_2893137; cat. # A32794, RRID:AB_2762834; and cat. # A32790, RRID:AB_2762833, respectively), were applied overnight at 4°C. On day 4, the slices were washed with PBS for 30 min three times, followed by nucleus staining with 4′,6-diamidino-2-phenylindole (DAPI) (1 μg/ml in PBS; Sigma Aldrich, UK; cat. # D9542) for 10 min. Then the slices were placed on glass microscope slides and a droplet of Vectashield fluorescent mounting medium (Vector Laboratories cat. # H-1000, RRID:AB_2336789) was applied to each slice. Coverslips were mounted and sealed and the slides were kept at 4°C until imaging.

### Imaging and image analysis

#### Rat glial precursor cell cultures

The automated microscope imaging system Arrayscan XTI (Thermo Fisher Scientific) was used for imaging. Images were acquired at 485 nm for GalC and 386 nm for nuclei using a 10x objective, to a total of 21 fields of view per well. Image analysis was carried out using the target activation bioapplication of the HCS Studio™ Cell Analysis software (Thermo Fisher Scientific, UK). The data obtained from each well were the mean number of nuclei (an index of the total number of cells) and GalC-positive cells, which allowed computing the number of GalC-positive cells per well as a percentage of the total number of cells. Then, for each culture, the mean of each well was normalized to and expressed as a relative percentage of the plate-averaged value of the vehicle-treated control wells.

#### Oligodendrocyte-enriched cultures

Ten images at 10x magnification were captured at 485 nm (FITC channel, MBP-positive cells) and 358 nm (DAPI channel, nuclei), at identical locations on each stained cover glass, with a Leica DM4000 epifluorescence microscope (Leica Microsystems UK Ltd., Milton Keynes, UK) interfaced with the Metamorph^®^ microscopy automation and image analysis software (Molecular Devices, LLC, San Jose, CA, USA). Cell quantification was performed with the ImageJ software (Rasband, W.S., NIH, Bethesda, Maryland) using scripts developed by Prof. John V. Priestley. The data obtained from each field of view were the number of nuclei and MBP-positive cells bearing membranous sheaths (which was computed as a percentage of the total number of cells per field of view and then normalized to and expressed as a relative percentage of the fields of view-averaged value of the vehicle-treated control wells), and the area covered by the cells bearing sheaths.

#### Organotypic slice cultures

The slices were imaged using a Zeiss LSM 710 confocal microscope equipped with the ZEN 2.1 software (Zeiss, Cambridge, UK). Twenty fields of view were arbitrarily positioned within each slice to cover the full slice and were imaged with a 40 × objective. Image analysis was carried out with the ImageJ software using dedicated scripts developed by Prof. John V. Priestley. The immunostaining derived either from MBP or calbindin staining alone, or the co-localization of MBP and calbindin, were thresholded out and the area covered by the staining was quantified. For Iba1 staining, the number of Iba1-positive cells was computed as a percentage of the number of DAPI-positive nuclei. The size of microglial cells was computed by using the cell size analysis function of ImageJ, with a minimum of 60 cells sampled across all slices in each treatment group.

### Statistical analysis

Statistical analysis was performed using GraphPad Prism 9 (GraphPad Software Inc., San Diego, CA, USA). The d’Agostino–Pearson test was used to check the normality of all data sets before running any statistical test. All data sets were plotted as mean ± S.E.M. for consistency. The level of significance was set at *P* < 0.05. No exclusion criteria were pre-determined, no test for outlier data was conducted, and all data points were included for analysis. A summary of the main experimental conditions and all statistical tests performed for every experiment, with the list of the corresponding figures, is given in Supplementary Table 1.

#### Rat glial precursor cell cultures

For each biological repeat and each dose, the values obtained from the three wells were averaged and used for analysis (*N* = 3 biological repeats). Data were analyzed with a two-way ANOVA followed by Šídák’s multiple comparisons test.

#### Oligodendrocyte-enriched cultures

In this experiment, data were obtained from only two biological repeats (originating from two cover glasses per dose from one biological repeat and one cover glass per dose from the other repeat), therefore, means obtained in each biological repeat could not be used as individual data. Rather, for each dose, the average of the three cover glasses was computed at each of the 10 sampled locations (N = 10 imaging locations), except for dose 2.4 mM where one cover glass had been mishandled and had to be discarded from the experiment. Depending on the outcome of the normality tests, data were analyzed with a one-way ANOVA followed by Dunnett’s multiple comparisons test, or a Kruskal–Wallis test followed by Dunn’s multiple comparisons test.

#### Organotypic slice cultures

For each experiment, 10–11 slices, obtained from 2 to 3 biological repeats, were analyzed per treatment group by an experimenter who was blind to the groups. The sample size was estimated from pilot studies. The values obtained from the 20 fields of view were averaged for each slice (*N* = 10–11 slices) and used for analysis. For cell size analysis of microglia, sampling was done evenly across all slices (*N* = 60 cells). Depending on the outcome of the normality tests, data were analyzed with an unpaired, two-tailed *t*-test, a one-way ANOVA followed by Dunnett’s multiple comparisons test, or a Kruskal–Wallis test followed by Dunn’s multiple comparisons test. Sets of data that were collected at two different time points were analyzed with a two-way ANOVA followed by Šídák’s multiple comparisons test.

## Results

### Cell cultures

We detected no toxic effect of high Phe concentrations in the cell culture models we tested – a culture of purified rat OL progenitor cells (Supplementary Figure 1) and a dissociated OL-enriched culture derived from P2 mice (Supplementary Figure 2). Rather surprisingly, high Phe concentrations added to the culture medium of purified rat OL progenitor cells appeared to promote the maturation of the cells after 4 or 7 days of application, which was most evident at the highest, non-physiological concentrations of 5 and 10 mM, where significantly increased numbers of GalC-positive cells were seen compared to the control group (Treatment, *F*(3,16) = 8.19, *P* < 0.01; Šídák’s multiple comparisons test, Phe 5 mM *vs* Phe 0.2 mM, *P* < 0.05; Phe 10 mM *vs* Phe 0.2 mM, *P* < 0.001; Supplementary Figure 1). A similar result was seen in OL-enriched cultures, where the non-physiological concentration of 4.8 mM also resulted in a significant increase in the number of MBP-positive cells bearing a membranous sheath (*H*(3) = 7.96, *P* < 0.05, Supplementary Figure 2), resulting in a mean 29% increase in the total area covered by these cells (Treatment, *F*(3,36) = 0.95, n.s.), but this increase in area was not due to an increase in the production of membranous sheaths per cell, which was unaffected by the treatment (Supplementary Figure 2).

### Organotypic slice cultures

#### Experiments 1 and 2: The effect of phenylalanine dose and exposure time on myelin

**FIGURE 2:**
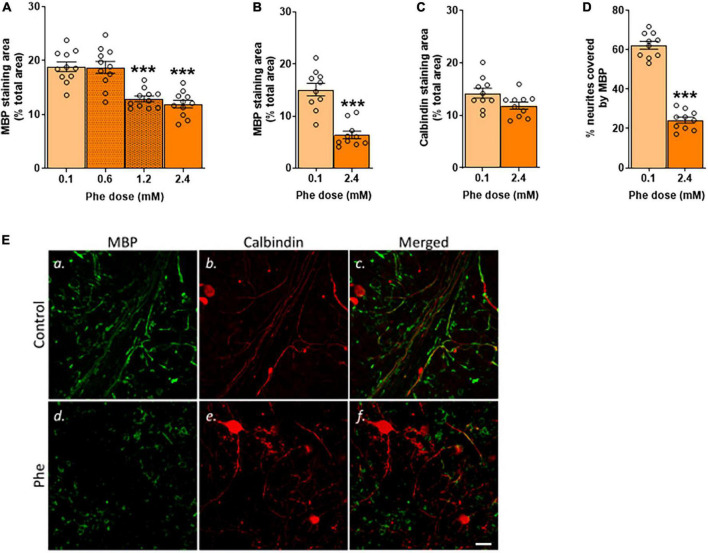
Only after 21 DIV were the highest doses of phenylalanine deleterious to MBP staining. **(A)** Cerebellar slices were incubated in control or Phenylalanine (Phe)-supplemented medium for 21 days *in vitro* (DIV) at 0.1, 0.6, 1.2, and 2.4 mM. Results are presented as the area covered by myelin basic protein (MBP) staining as % of the total area of the field of view. Data are plotted as Mean ± SEM of *N* = 10 slices/treatment group. **(B–D)** Cerebellar slices were incubated for 21 DIV in a control medium (0.1 mM Phe) or a medium containing 2.4 mM Phe. The graphs show the results of the immunohistochemistry quantification: **(B)** Mean area covered by MBP staining as % of the total area of the field of view; representative images are shown in panels (**E**a,d); **(C)** Mean area covered by calbindin staining as % of the total area of the field of view; representative images are shown in panels (**E**b,e); **(D)** % of neurites stained for MBP; representative images are shown in panels (**E**c,f). Data are plotted as Mean ± SEM of *N* = 10 slices/group. Scale bar = 20 μm. Statistical symbols indicate significance at ^***^P < 0.001.

First, we assessed the effect of increasing concentrations of Phe on myelin using MBP as a marker (Figure 1A). The lowest Phe concentration (0.6 mM) did not affect MBP staining but a significant reduction in the area covered by MBP was seen after 21 DIV of treatment with 1.2 and 2.4 mM Phe, compared to the control group (Figure 2A).

We then tested the highest Phe concentration (2.4 mM) over different durations of exposure (7, 10, or 21 DIV –Figure 1B) to assess the earliest time point at which we could detect a reduction in MBP staining. There was no detectable effect at 7 DIV (data not shown) or 10 DIV (Supplementary Figure 3) but a significant reduction was seen at 21 DIV; therefore, in subsequent experiments, slices were incubated with 2.4 mM Phe for 21 DIV. We confirmed that these conditions resulted in a highly significant, over twofold reduction in MBP signal (*t*(18) = 6.16, *P* < 0.001, Figures 2B,Ea,d) and in a trend toward a decrease in calbindin staining (*t*(18) = 2.03, *P* = 0.06, Figures 2C,Eb,e). The percentage of neurites stained with MBP was also significantly decreased, showing an almost threefold reduction compared with control slices (*t*(18) = 15.2, *P* < 0.001, Figures 2D,Ec,f).

#### Experiment 3: The effect of stopping high phenylalanine on remyelination

**FIGURE 3:**
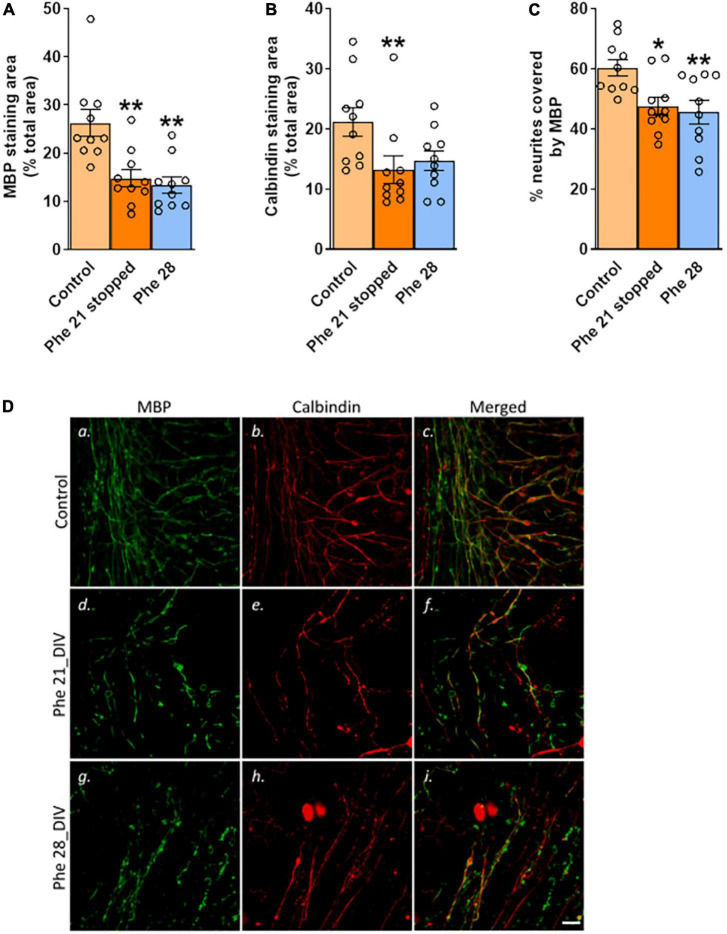
Stopping high phenylalanine for 7 days after 21 DIV of exposure did not result in detectable remyelination. Cerebellar slices were incubated for 21 or 28 days *in vitro* (DIV) in a control medium or a medium containing phenylalanine (Phe) at 2.4 mM. At 21 DIV, the Phe-enriched medium was replaced with the control medium (0.1 mM Phe) in one of the Phe-supplemented groups (called “Phe 21 stopped”), whereas the other Phe-exposed group was kept in the Phe-enriched medium for another 7 DIV (“Phe 28”). The graphs show the results of the immunohistochemistry quantification: **(A)** Mean area covered by myelin basic protein (MBP) staining as % of the total area of the field of view; representative images are shown in panels (**D**a,d,g); **(B)** Mean area covered by calbindin staining as % of the total area of the field of view; representative images are shown in panels (**D**b,e,h); **(C)** % of neurites stained for MBP; representative images are shown in panels (**D**c,f,i). Data are plotted as Mean ± SEM of *N* = 10 slices/group. Scale bar = 20 μm. Statistical symbols indicate significance at *P < 0.05 and ^**^P < 0.01.

Keeping the slices for 7 days in the control medium after 21 DIV of exposure to 2.4 mM Phe (Figure 1C) did not result in detectable remyelination. Whether 2.4 mM Phe was stopped after 21 DIV and followed by a further 7 days of culture in the control medium, or was continued until 28 DIV, the areas stained for MBP (*H*(2) = 12.8, *P* < 0.01, Figures 3A,D,a,d,g) and the levels of co-staining of MBP with calbindin (*F*(2,27) = 6.0, *P* < 0.01, Figures 3C,Dc,f,i) had significantly decreased over this period, compared to the control group, to final levels that were equivalent between the two treatment groups (MBP staining area, mean 44% decrease, Phe 21 stopped vs. 49% decrease, Phe 28, Figures 3A; % neurites covered by MBP, mean 21% decrease, Phe 21 stopped *vs* 24% decrease, Phe 28, Figure 3C). A decrease in calbindin staining area compared to control levels (*H*(2) = 8.5, *P* < 0.05, Figures 3B,Db,e,h) was noted but was only statistically significant in the group where 2.4 mM Phe had been stopped at 21 DIV (Dunn’s multiple comparisons *post-hoc* test, Phe 21 stopped *vs* control, *P* < 0.01; Phe 28 *vs* control, *P* = 0.12).

We checked, in a control experiment, that demyelination and remyelination were possible in our OSC model by exposing the slices to a medium supplemented with the bioactive lipid lysolecithin, known to induce demyelination in cerebellar slice cultures (Birgbauer et al., 2004). Two days after switching back to the fresh control medium, the percentage of neurites covered by MBP was significantly decreased in the slices treated with lysolecithin (*Mdn* = 55.1, controls; *Mdn* = 29.3, lysolecithin; *U*(*N*_control_ = 7; *N*_Phe_ = 6) = 4.0, *P* < 0.05, Supplementary Figure 4), corresponding to a mean 43% decrease from the control values, showing severe demyelination. But 11 days later, at 21 DIV, while the percentages of neurites covered by MBP had increased in both treatment groups over this period (DIV, *F*(1,19) = 13.38, *P* < 0.01, Supplementary Figure 4), this relative increase amounted to to 30% for the control slices but to a dramatic 126% for the slices initially treated with lysolecithin, mean values of both treatment groups being now undistinguishable at this time point (% of neurites covered by MBP at 21 DIV relative to mean control values at 10 DIV, 130.0 ± 20.85%, controls; 128.1 ± 18.68%, lysolecithin, Supplementary Figure 4), demonstrating that a very active remyelination process was occurring in the slices that had been previously demyelinated by lysolecithin.

#### Experiment 4: The effect of high phenylalanine on microglial activation

**FIGURE 4:**
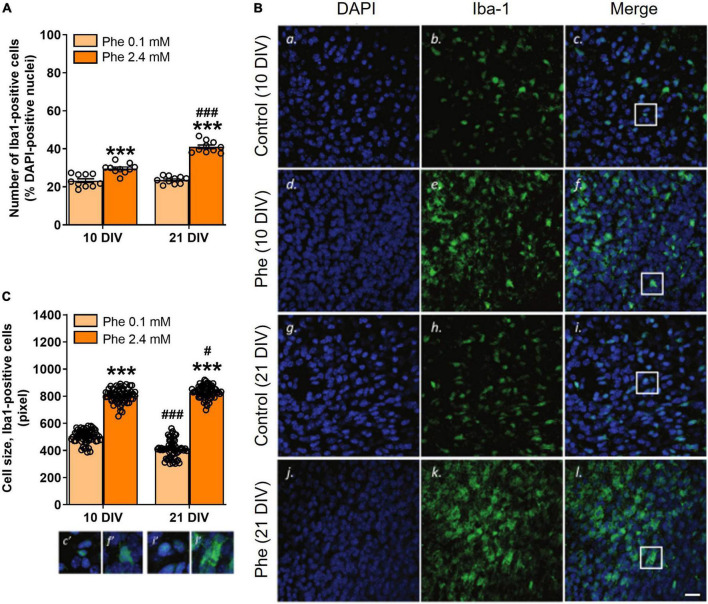
Reactive microglia were seen as early as 10 DIV following continuous treatment with high phenylalanine. Cerebellar slices were incubated for 10 or 21 days *in vitro* (DIV) in a control medium or a medium containing phenylalanine (Phe) at 2.4 mM. The graphs show the results of the immunohistochemistry quantification. **(A)** Number of Iba1-positive cells as % of DAPI-positive nuclei from 20 fields of view per slice and 10 slices for each treatment group (*N* = 10); representative images are shown in panels (**B**c,f,i,l); **(C)** Size analysis at 10 and 21 DIV of 60 microglial cells sampled across 10 slices in each treatment group; corresponding images are shown in panels (**B**c,f,i,l). Data are plotted as Mean ± SEM of *N* = 10 slices/group. The insets (c’, f’, i’, l’) show details of the colocalization of the two stainings and of the morphological differences between treatment groups and time points. Note the different morphologies (amoeboid vs. elongated) and sizes of the cells as a function of their activation state. Scale bar = 20 μm. Statistical symbols indicate significance at ***P < 0.001 of Phe-treated slices vs. control slices and at ^#^*P* < 0.05 and ^###^*P* < 0.001 of 21 DIV vs. 10 DIV.

The analysis of slices stained with the microglia/macrophage marker Iba1 showed that treatment with 2.4 mM Phe for 10 or 21 DIV (Figure 1D) resulted in higher numbers of Iba1-positive cells (Treatment, *F*(1,36) = 180.8, *P* < 0.001, Figure 4A), an effect that increased over time (Treatment x DIV, *F*(1,36) = 38.7, *P* < 0.001; Šídák’s multiple comparisons test, DIV, control, *P* = 0.94; Phe, *P* < 0.001; Figures 4A,B). Cell morphology analysis showed that the mean size of somata of Iba1-positive cells was significantly larger after Phe treatment than that of cells from control slices, at both time points (Treatment, *F*(1,236) = 2,669.5, *P* < 0.001, Figure 4C). Mean cell size values in Phe-treated slices significantly increased with incubation time while they significantly decreased, from 10 to 21 DIV, in slices incubated with the control medium (Treatment x DIV, *F*(1,236) = 71.9, *P* < 0.001; Šídák’s multiple comparisons test, DIV, control, *P* < 0.001; Phe, *P* = 0.05, Figure 4B).

## Discussion

White matter abnormalities represent a key feature of PKU that is detectable even in individuals who are diagnosed early after birth. The underlying mechanisms of PKU remain unclear.

Phenylketonuria is a heterogeneous condition; in different PKU patients, the WM pathology may represent a failure of myelin formation, or progressive damage to myelin and failure to fully remyelinate, or a combination of several processes. WM deficits can be reproduced in rats or mice either through sustained exposure to high intake of Phe or genetic manipulations but, so far, *in vivo* models have not been conclusive on whether Phe is directly toxic to OLs, the specialized glial cells that produce myelin and ensheathe axons. It is also unclear whether high Phe creates a hostile environment for both neurons and OLs, or whether the GM and WM pathologies follow a sequence, i.e., i) neuronal development is affected first, specifically axonal maturation, triggering hypomyelination or destabilizing mature neurites, followed by a breakdown of myelin membranes, or ii) the initial impact is on OLs, with the resulting dysmyelination impairing neuronal growth and connectivity, ultimately further destabilizing OLs and driving demyelination and axonal demise. There is still a need for appropriate *in vitro* models to study these questions and understand the pathophysiology of this disease. The purpose of our study was to use *in vitro* platforms of varying complexity to allow the application of controlled Phe concentrations directly onto cells or brain tissue to study the details of cellular toxicity of high Phe, which is not possible using *in vivo* PKU models. We included a cerebellar OSC model platform, which has the added advantage to allow studying the impact of high Phe under conditions of relative preservation of neural circuitry, which cannot be achieved with cell culture models.

The cerebellar OSCs retain the original three-dimensional aspect of the tissue, with all major cell types that are known to interact in the native brain tissue matrix being present. Because the slices were obtained from mouse pups at P8, myelination can be considered as almost complete from 4 DIV onward (equivalent to P12 cerebellum), and the slices would be equivalent to “mature cerebellar OSCs” by 15 DIV (P23 cerebellum), with most axons being in an adult state of myelination at this stage (Foran and Peterson, 1992). In this model, we present evidence that high Phe concentrations are significantly toxic to myelin after long exposure times. It was not until 21 DIV (i.e., P29 cerebellum) that Phe at concentrations ≥1.2 mM resulted in a significant decrease in MBP, an index of the presence of myelin, but no significant intrinsic impact was detected on neurites following this period of exposure. The fact that sustained exposure of myelinated tracts to high Phe is critical to observe its toxicity is an important point to consider since, in the 1970s, many practitioners believed that high Phe would no longer affect the brain once it was fully developed and, as a result, many supported discontinuing the diet past 6 years of age (Berry et al., 2013). When looking at the lifetime exposure to Phe of early- and continuously treated PKU patients, the prolonged exposure to elevated Phe and also the variability of Phe levels are the best predictors of widespread microstructural WM integrity compromise (Hood et al., 2015). Phe variability is also a stronger predictor of cognitive performance than any other index of Phe control (Hood et al., 2014). It will thus be interesting in future studies to model such variability of Phe levels, which is associated with the genotype of PKU patients and correlates significantly with the predicted residual enzyme activity (Rupp et al., 2001).

The details of the MBP-driven formation of the major dense line –the 3 nm compartment between cytoplasmic leaflets of stacked myelin membranes mostly occupied by MBP– suggest that the stability, structure, and mode of association of MBP with the membrane need to be considered for a full understanding of the pathophysiological mechanisms underlying demyelination (Raasakka et al., 2017). Therefore, the apparent absence of effect of high Phe on MBP staining at shorter time points in the OSCs does not necessarily imply functional integrity of myelinated tracts. In a genetic mouse model of PKU, the Pah^enu2^ mice, WM tracts show abundant MBP in mature adults but the expression of MBP isoforms is aberrant (Dyer et al., 1996). That the protein had retained its antigenicity but not its full functionality after high Phe cannot be excluded in our study. It might be of interest to investigate in future experiments if other myelin proteins that function similarly to MBP, synergistically or competitively, such as myelin protein zero and myelin protein P2 [references in Raasakka et al. (2017)], are affected by high Phe during the phase of active myelination.

An important observation in our model is that once the toxic effect of Phe was fully developed, exposure to the control medium did not lead to overt remyelination, as opposed to what was seen in our control experiment with lysolecithin. OLs have a key role in the myelinating process, with most OPCs differentiating into pre-myelinating and then mature and myelinating OLs, but a small amount remains immature or in a quiescent state n both GM and WM as a potential source of mature OL replacement in response to a demyelinating disorder such as PKU (Bergles and Richardson, 2016; Ferreira et al., 2020). Following injury or disease, OPCs accelerate their cell cycle for increasing the production of OLs to replace lost myelin, which is seen in the majority of the differentiated progeny, but they can also give rise to glial fibrillary acidic protein (GFAP)-expressing astrocytes, and following some types of injury (e.g., freeze-thaw damage to the cerebral cortex), OPCs have been shown to generate exclusively protoplasmic astrocytes rather than OLs (Zhu et al., 2012). The density of OPCs is reduced in demyelinated lesions of the post-mortem brain of MS patients [references in Bergles and Richardson (2016)], indicating disruption of their homeostatic control. It is thus legitimate to ask whether prolonged high Phe is toxic to the OPCs or modifies their phenotype toward an inability to proliferate and differentiate into myelinating OLs in response to the demyelinating insult.

Alternatively, we cannot rule out that 1 week of exposure to the control medium was too short a time in our model for recovery of myelination following Phe toxicity –in the lysolecithin model, remyelination was achieved after 13 days. Dyer et al. (1996) have shown that in humans and mice affected by PKU the white matter is gliotic in the cerebrum and cerebellum, and OLs show an altered, non-myelinating phenotype expressing GFAP but the cells are viable. It would be of interest to demonstrate if these cells can revert their phenotype to a myelinating one, and that both proliferation of OPCs and remyelination occur following the control of Phe levels, and ascertain the needed timeframe of these events if they occur. In addition to OL cytotoxicity and the inhibition of enzymes involved in myelination, high Phe also results in aberrant DNA methylation, resulting in putative gene silencing and abnormal protein expression -which is known to happen, for instance, for neural cell adhesion molecule (NCAM) and MBP [reviewed in Ferreira et al. (2020)]. It has been suggested that the severity and reversibility of chronic demyelination in early diagnosed and continuously treated patients might be linked to adherence to the diet and the profile of metabolic control [references in Borges et al. (2022)]. Our model will be useful in future research to look further into the details of the mechanisms involved in the reversibility of the demyelination, or its failure. Focusing on the behavior of OPCs and OLs under high Phe and following the normalization of Phe levels is warranted.

An additional element that will need to be taken into consideration for future research is the complex bidirectional interactions that are known to exist between neurons and oligodendroglia for appropriate CNS myelin targeting and optimal functioning of WM circuits (Almeida, 2018; Mazuir et al., 2021). A significant decrease in calbindin staining area was seen in the group where Phe was corrected back to normal from 21 DIV onward (Figure 3B). Such an effect was not found when slices were fixed immediately after 21 DIV of treatment (Figure 2C). This may indicate a delayed toxic effect on neurites, which might impact the chances of successful remyelination. In contrast to chronic WM abnormalities, which have been well studied, less is known about GM changes in PKU patients. It is usually assumed that neurons and axons are spared (Allen and Kirk, 1992; Dyer et al., 1996) but GM changes, e.g., reduction in dendritic arborization, synaptic loss, decreased plasticity and excitability, and neurodegeneration, have also been reported (Dyer, 1999; Huttenlocher, 2000; Hörster et al., 2006; Horling et al., 2015; González et al., 2016). A study in adult PKU untreated patients with intellectual disability showed widespread disruption affecting both GM and WM (Bauman and Kemper, 1982). Neuronal loss, along with reduced size of surviving neurons and decreased dendritic processes of Purkinje cells, was reported in tissue from an untreated patient (Kornguth et al., 1992). By contrast, no gross changes in the numbers of neurons were found in the brains of the homozygous Pah^enu2^ mice (Dyer et al., 1996). In a mixed murine primary cortical culture exposed to 5 mM Phe for 3 weeks, a significant reduction in synaptogenesis was found but with viable cortical neurons or dendritic arborizations (Hörster et al., 2006). Our findings suggest that calbindin-positive neurites are relatively resilient to high Phe, although protracted damage may occur. Genetic deletion of calbindin is known to affect the function of the cerebellum, with a deleterious impact on the precision of motor coordination (Barski et al., 2003). Interestingly, it was reported in one study that even early treated PKU children have lower motor development (Nazi et al., 2014).

Microglial cells are known to present in an activated state in cerebellar OSCs (Dusart et al., 1997) and are involved in the constant phagocytic clearance of dead cells, protein aggregates, pathogens and accumulated debris, including myelin debris (Hanisch and Kettenmann, 2007; Neumann et al., 2009). We assessed the state of activation of these cells in control slices and after exposure to Phe. Following treatment with high Phe, the numbers of Iba1-positive cells significantly increased as early as 10 DIV; a larger increase was seen at 21 DIV, a time when the deleterious impact of high Phe on myelination was significant. Since microglial cell morphology and function are closely linked (Fernández-Arjona et al., 2017), we also assessed their activation state by measuring their size and through morphological qualitative observations. In control slices, a significant decrease in the mean size of Iba1-positive cells was seen with time spent in culture, consistent with a progressive decrease in their activation state following the establishment of the culture. This is in line with previously published work. In cerebellar OSCs from P10 rat pups (equivalent to P8 in mice), many cells die and numerous microglia/macrophages are activated during the first week of culture, but then the slices recover (Dusart et al., 1997). In our experiments, we found a large increase in the mean size of Iba1-positive cells in treated slices at both 10 and 21 DIV, compared to control slices, suggesting that increased microglial activation was present early after treatment, before any obvious signs of myelin loss were detected. In Phe-exposed slices, Iba1-positive cells presented as very dense aggregations of cells with large rounded somas and thick and short processes, an amoeboid phenotype characteristic of activated microglia. In control slices, cells were more sparsely distributed and presented with much smaller somas, some with thin processes. Activated cells showed cellular hypertrophy, a morphology that is in accord with that described in control rat cerebellar OSCs at 7 and 17 DIV (Dusart et al., 1997). Microglial cell somas decreased in size with time in control OSCs, as a sign of recovery and adaptation to the culture process. It is important to note that microglial activation is not ruled by a simple on/off mechanism and does not show a unique pattern (Fernandes et al., 2014), the cells present as a heterogeneous population that exists along a continuum where their acquired profile depends on the local environment (Joers et al., 2017). In addition, the reactivity of their phenotype (cellular hypertrophy and increased expression of several factors, including immune mediators) precedes their proliferation, which is thought to be a later event (Atallah et al., 2014).

To our knowledge, our observations identify for the first time a state of strong activation of microglial cells following sustained exposure of a slice of immature brain tissue to a high Phe concentration. In a previous study, the state of activation of microglia had been described in hippocampal sections from Pah^enu2^ mice. In this PKU model, the point mutation can be expressed on different genetic backgrounds, giving rise to either the black and tan brachyury (BTBR) PKU or the C57BL/6 PKU mouse model (van der Goot et al., 2019). A mildly enhanced activation was seen in the C57BL/6 PKU mice compared to their wild-type littermates but not in the BTBR PKU mice, where basal levels of activation were already high in wild-type counterparts, and significantly higher than in the C57BL/6 wild types (van der Goot et al., 2019). The authors hypothesized that this increased response in C57BL/6 PKU mice was related to a neuroprotective effect of microglia, which would support hippocampal function by maintaining a healthy balance between excitatory and inhibitory synapses, through microglia-mediated synaptic pruning. They did not discuss their findings from the perspective of an impact on the integrity of the WM. It is noteworthy that more hippocampus-dependent deficits have been reported in the model where hippocampal basal levels of microglial activation are already higher in the control wild type, the BTBR PKU mouse model (Bruinenberg et al., 2016). Nevertheless, in a study by Schlegel et al. (2016), Phe at 5 mM did not affect microglial phagocytic activity when applied for 1 h in primary microglial cultures obtained from whole brains of P3 C57BL/6 mice. When applied for 3 days to dissociated hippocampal neurons from C57BL/6 mice, this Phe concentration resulted in reduced dendritic arborization, with a biphasic dose-effect on dendrite branching (1 mM Phe increasing it but 5 mM decreasing it) and no effect on axonal growth. When organotypic hippocampal slices from C57BL/6 mice were treated with Phe at 1, 2, and 5 mM, a dramatic reduction in synapse density was seen but none of the doses affected microglia, as assessed by the intensity of Iba1 staining. Compared to what Dusart et al. (1997) and ourselves report in control cerebellar OSCs, the hippocampal microglia in Schlegel et al. (2016) from both control or treated slices resembled “surveying,” mildly activated cells –corresponding to the old terminology of “resting” microglia (de Miranda et al., 2017). Data from human and rodent tissues have shown that variability exists in cell density, expression of surface markers, and responsiveness to challenges between microglia from different brain regions [references in Joers et al. (2017)], and these regional differences in profiles are not simply dependent on the local environment, they are inherent to the microglial cells, as they are preserved *in vitro* (Ren et al., 1999; Xie et al., 2003; Joers et al., 2017). It is thus likely that the differences in the basal state of microglial activation that may exist between cerebellar and hippocampal slices are not fortuitous. Regional variations in the baseline expression profile of microglia might predict their responsivity, which may contribute to regional vulnerability and susceptibility of neurons (Doorn et al., 2015; Joers et al., 2017). An important point is that our results were obtained in an OSC model derived from an outbred strain, which adds another element of complexity, and thus potential variability, to the basal reactivity levels of microglia. This is important in a disease where there is a wide variability of WM damage under identical values of blood and brain Phe (van Wegberg et al., 2017).

Our results thus raise the possibility that high Phe in brain tissue leads to a state of enhanced activation of microglia that may play a critical role in the demyelination of WM tracts. However, the cause-effect relationship between (i) high Phe and microglial activation and (ii) microglial activation and demyelination, needs to be examined further. In future studies, it would be informative to experiment on OSCs obtained from BTBR wild-type mice and study the effect of high Phe in these mice, whose microglial activation is already enhanced at the basal level, compared to C57BL/6 wild types, and also on slices from BTBR PKU mice. BTBR mice have a higher proportion of MHC class II-expressing microglia than C57BL/6 mice, but also overall aberrant immune activities, suggestive of an autoimmune profile (Heo et al., 2011), and they present with clear hypomyelination as adults (Wahlsten et al., 2003). Evidence of neuroinflammation in the brain of PKU patients is still controversial. However, as recently discussed by Ferreira et al. (2020), the apparent discrepancy of most findings reported so far may stem from the fact that neuroinflammation is only seen in the brain of patients who were diagnosed at a late stage, as opposed to patients who were diagnosed by newborn screening programs and whose Phe levels had thus been controlled from early stages of development. Our results support this contention since, in our experiment, significant increases in Iba1-positive cell numbers were seen at a time where myelination can be considered as almost complete (10 DIV) to complete (21 DIV), corresponding to a stage where exposure to high Phe had been continuous from an immature to an adult state of myelination.

Our observations in the cerebellar OSC model open new avenues for investigation of the impact of high Phe concentrations on the CNS and for generating new insights into the mechanisms underlying WM pathology in PKU. We detected significant toxic effects of Phe at concentrations that are relevant to plasma Phe levels seen in PKU patients. We did not measure the Phe content in tissue to get an indication of the Phe concentration that was reached in slices. In PKU patients, brain Phe can reach levels between 16 and 44% of plasma Phe levels (Pietz et al., 1999; Cleary et al., 2013), but the relationship between brain and Phe levels varies significantly between individuals. OSCs were cultured in a medium whose composition is not similar to that of the cerebrospinal fluid and, at present, it is also not possible to infer what is the flux of Phe from the medium into the slices and how the Phe concentrations that were reached in slices compare to these found in the brains of patients.

Like all *in vitro* models, cerebellar OSCs cannot reproduce all dimensions of the PKU pathology and its sheer complexity, which involves a failure of human neurodevelopment and various other processes (Borges et al., 2022). To understand the molecular parameters driving PKU and fully recapitulate the pathophysiology of the condition, Borges et al. (2022) recognize that there is a need for using both advanced humanized culture models and models containing multiple cell types. *In vitro* models using simply cells, i.e., pure OL cultures, mixed glial cell cultures, or neuronal-glial cultures, may have failed to show a deleterious effect of Phe on myelin because of the lack of cell-cell interactions and the absence of key cellular players that may mediate the impact of Phe on OPCs and OLs. The results we obtained in two simpler, cellular models support this idea and suggest that the effect of high Phe may involve a complex interplay between various cell types that can be studied in a system that preserves the three-dimensional structure that is reminiscent of *in vivo* brain conditions (Humpel, 2015). Bergles and Richardson (2016) offer a very thorough description of the complexity of the temporal and spatial control of the proliferation and differentiation of the OPCs *in vivo*, and of the other cells and molecular actors involved in this process. For instance, neighboring neurons and astrocytes are important for the survival and proliferation of OPCs (and enabling their movement) through the secretion of PDGF-AA, but also are the extracellular matrix molecules (such as the NG2 proteoglycan and tenascin-C) and their integrin receptors, which actively co-participate in the maintenance of the OPCs mitogenic activity. At a minimum, these elements should be present in a PKU model testing the mechanisms of Phe toxicity. In addition, and as is seen in other demyelinating diseases (Peterson and Fujinami, 2007), our results suggest that the activation of microglial cells may play a significant role in the demyelinating insult, but this aspect is still underestimated and the role of the neuroinflammation and microglia in the pathophysiology of PKU needs to be investigated further. An additional level of cellular complexity involves the blood-brain barrier since Phe requires the L-type amino acid carrier to gain access to the brain. High Phe concentrations can impair, through competition, the transport of the monoamine precursors tyrosine and tryptophan (Williams et al., 2008), affecting the synthesis of serotonin and dopamine in the CNS. Competition for this transporter may also affect cerebral protein synthesis, including enzymes involved in the biosynthesis of cholesterol. Evidence was found in immature chicks that high Phe concentrations inhibit these enzymes (Castillo et al., 1988), negatively impacting myelin but indirectly because of the importance of cholesterol for myelination. Future studies will need to integrate and explore these aspects, which would benefit from the development of promising novel three-dimensional models, such as brain-vascular organoid systems (Borges et al., 2022), for instance, but cannot be resolved with our OSC model.

## Data availability statement

The raw data supporting the conclusions of this article will be made available by the authors, without undue reservation.

## Ethics statement

Ethical review and approval was not required for the animal study because this is an *in vitro* study.

## Author contributions

OT-Z: methodology, investigation, formal analysis, visualization, and writing – original draft. PP: methodology, investigation, formal analysis, validation, visualization, writing – review and editing, and final draft. PS and AK: methodology and investigation. JMV: writing – review and editing. AM-T: funding acquisition, conceptualization, methodology, supervision, and writing – review and editing. All authors contributed to the article and approved the submitted version.

## Abbreviations

ACSF: artificial cerebrospinal fluid
BSA: bovine serum albumin
BTBR: black and tan brachyury
CNS: central nervous system
DAPI: 4′,6-diamidino-2-phenylindole
DIV: days *in vitro*
DMEM: Dulbecco’s modified eagle’s medium
FBS: fetal bovine serum
GalC: galactocerebroside
GFAP: glial fibrillary acidic protein
GM: gray matter
MBP: myelin basic protein
MEM: minimum essential medium eagle
MGCM: mixed glial cell culture medium
MGCMIns: insulin-containing MGCM
OL: oligodendrocyte
OPCs: OL precursor cells
OSC: organotypic slice culture
P: postnatal day
PAH: phenylalanine hydroxylase
PBS: phosphate-buffered saline
PDGF-AA: platelet-derived growth factor-AA
Phe: phenylalanine
PKU: phenylketonuria
RGPCs: rat glial precursor cells
RRID: research resource identifier
WM: white matter.
