# Supplementary material for: High phenylalanine concentrations induce demyelination and microglial activation in mouse cerebellar organotypic slices
Source: Front Neurosci. 2022 Sep 29;16:926023. doi: 10.3389/fnins.2022.926023 (PMC9559601; doi:10.3389/fnins.2022.926023)
Supplement: Supplementary file 1 [file Data_Sheet_1.pdf]

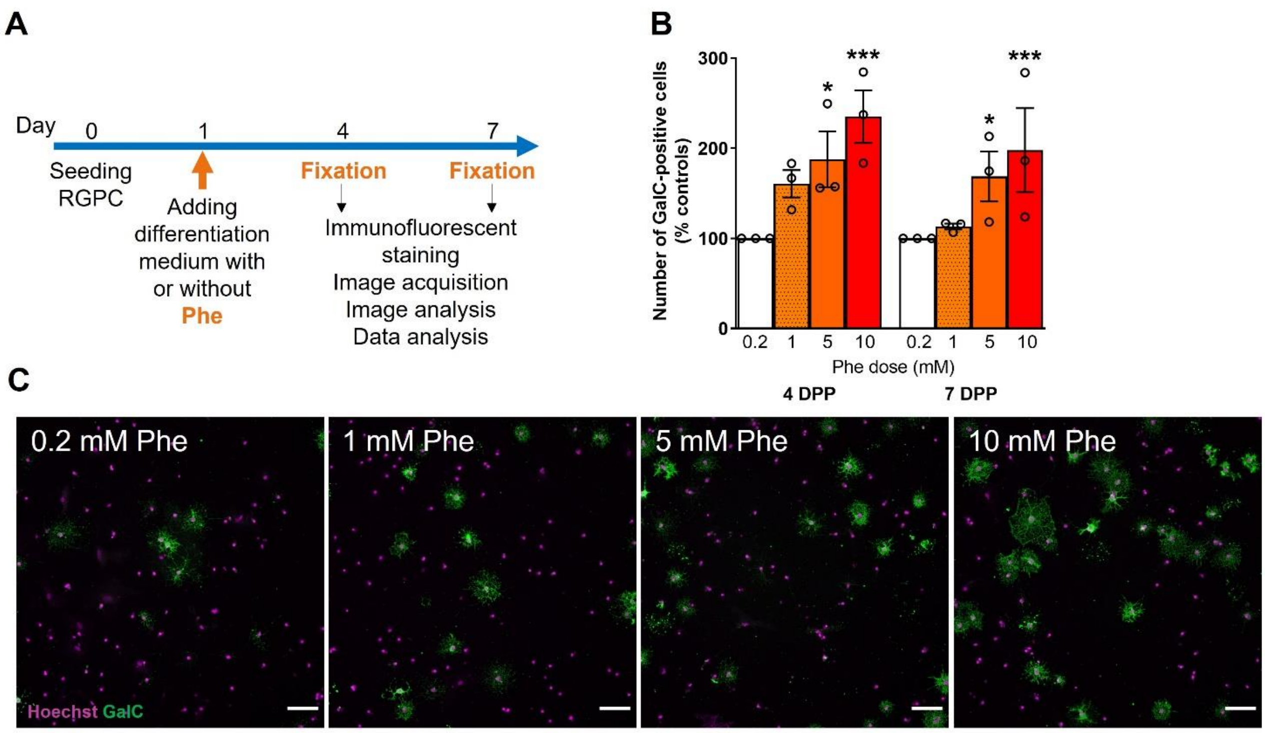

Figure S1. Direct application of high phenylalanine to rat glial precursor cells was not toxic but promoted the differentiation of the cells towards mature oligodendrocytes

High Phe was not toxic to oligodendrocytes cultured from rat glial precursor cells up to the non-physiological concentrations of 5 and 10 mM, after either 3 or 6 days of exposure; rather, the non-physiological concentrations resulted in increased numbers of GalC-positive cells. (A) Study timeline. (B) The relative number of GalC-positive cells (as a percentage of the mean of the control wells) after treatment with 4 different Phe concentrations (0.2, 1, 5, and 10 mM) for either 3 or 6 days (corresponding to cells fixed at 4 and 7 DIV, respectively). Data are plotted as Mean ± SEM of $N = 3$ culture replicates. (C) Representative images for each treatment, at 10x magnification, of cells fixed at 7 DIV. Scale bar = 100 µm.

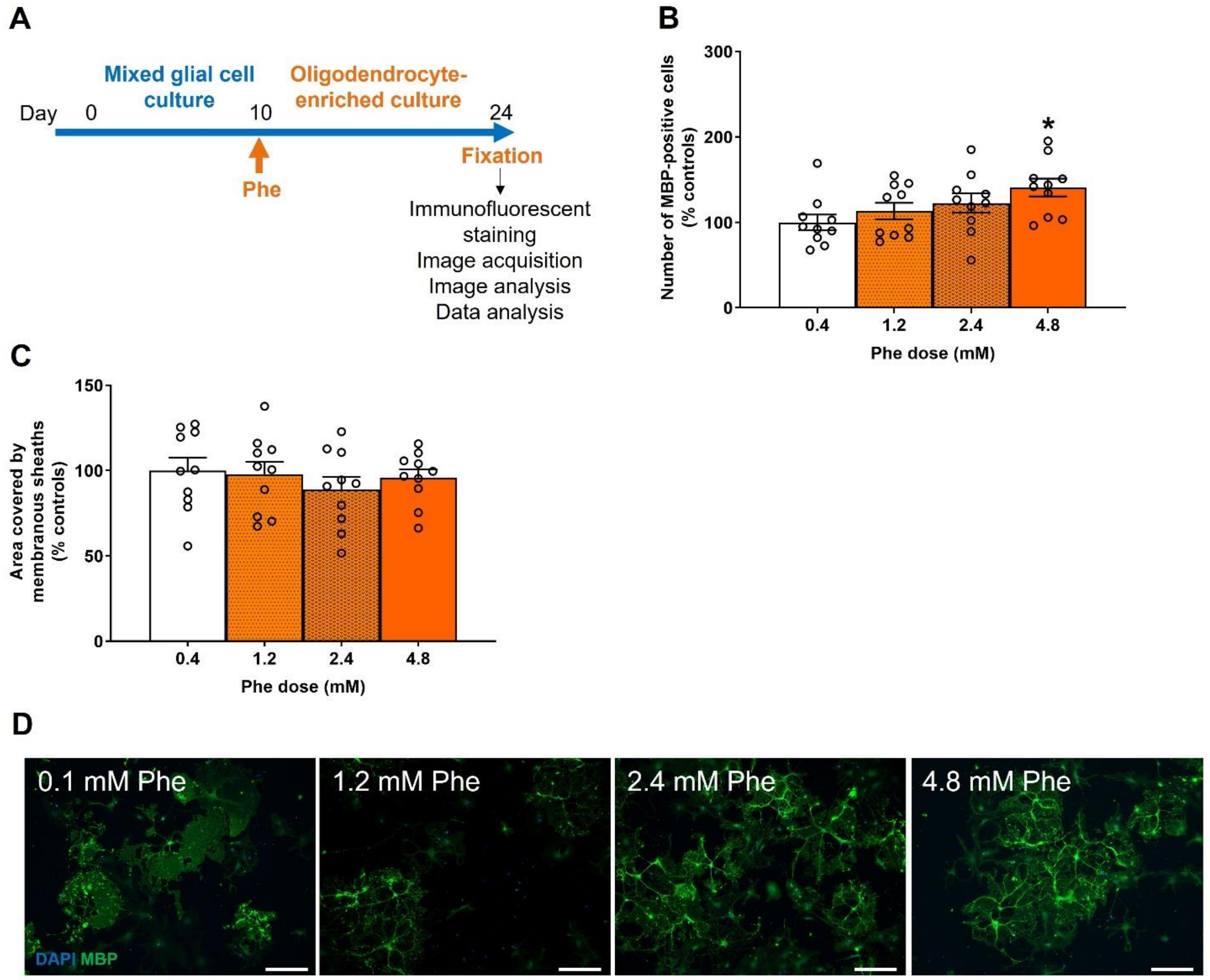

Figure S2. Direct application of high phenylalanine to mouse oligodendrocytes was not toxic but promoted the differentiation of the cells towards mature oligodendrocytes.

High Phe was not toxic to oligodendrocytes in oligodendrocyte-enriched cultures up to the non-physiological concentration of 4.8 mM. (A) Study timeline. (B) The relative number of MBP-positive cells with membranous sheaths (as a percentage of the mean of the control wells) after treatment with 4 different Phe concentrations (0.4, 1.2, 2.4, and 4.8 mM). (C) The area covered by MBP-positive cells bearing membranous sheaths per cell, relative to control values. Data are plotted as Mean ± SEM of N = 10 fields of view. (D) Representative images for each treatment at 20x magnification. Scale bar = 100 µm.

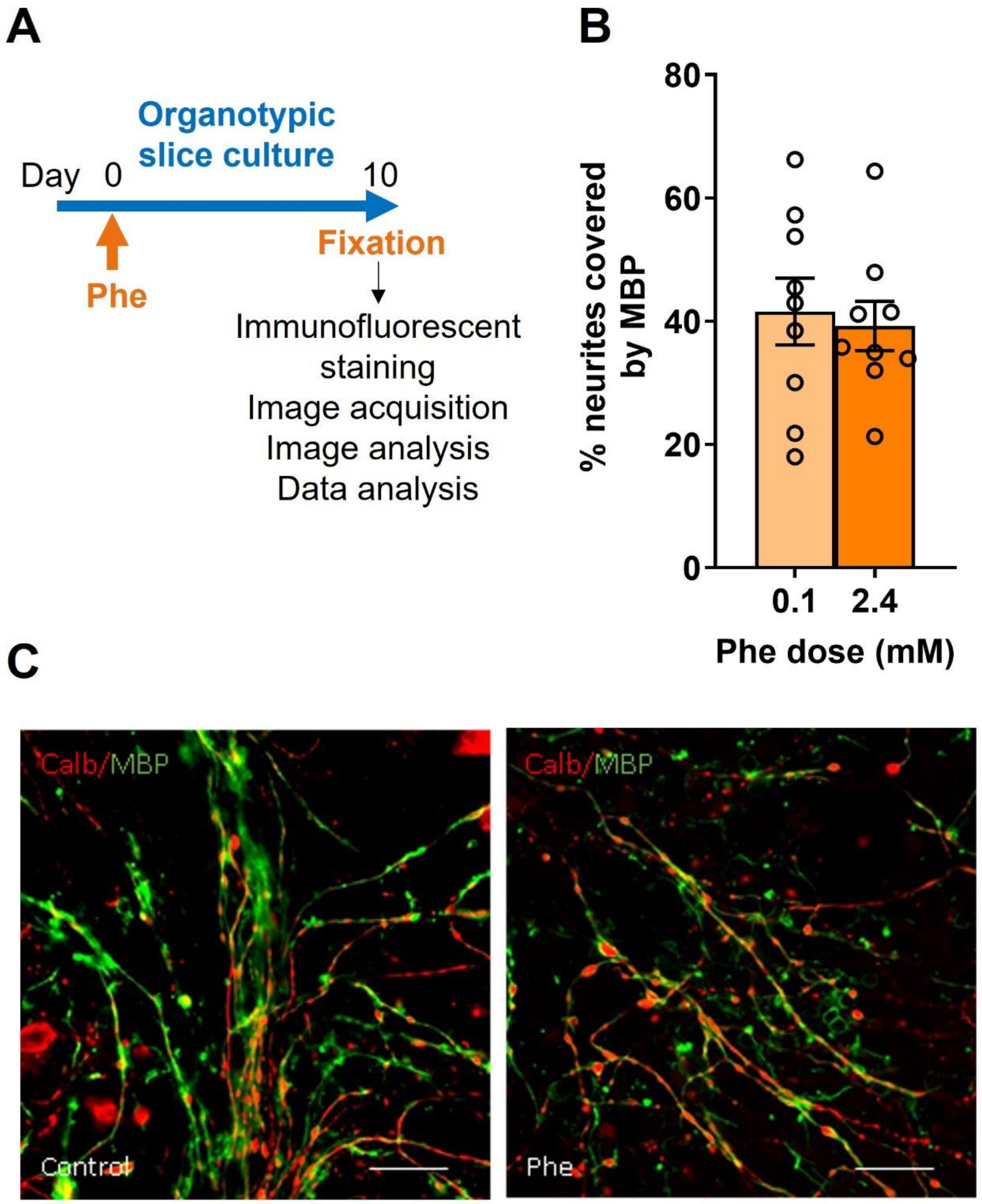

Figure S3. High phenylalanine applied to cerebellar organotypic slices did not reduce the number of neurites covered by MBP at times earlier than 21 DIV.

After either 7 DIV (data not shown) or 10 DIV ((B) and (C)), the numbers of neurites covered by MBP were similar in the control and treatment groups. (A) Study timeline. (B) % of neurites stained for MBP. (C) Representative images. Data are plotted as Mean ± SEM of N = 10 slices/group. Scale bar = 20 µm.

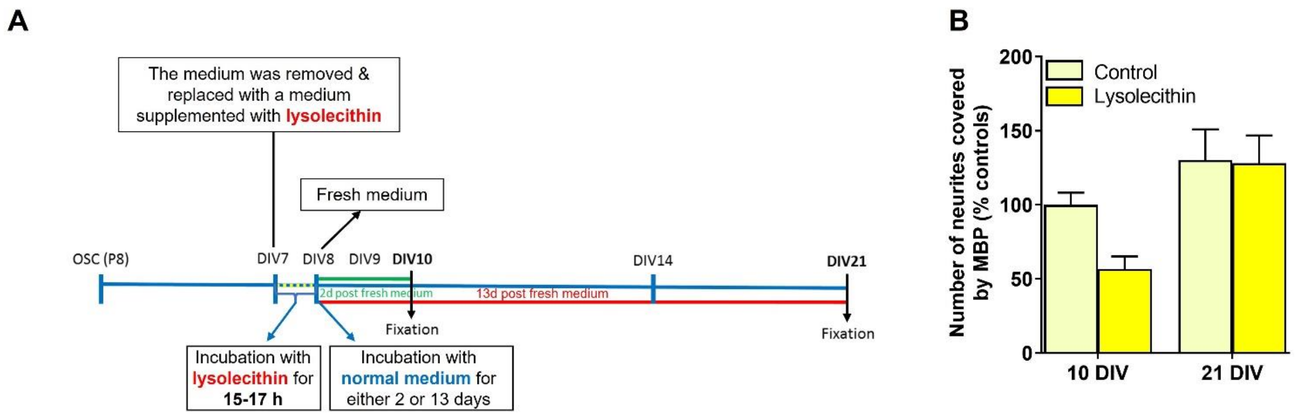

Figure S4. Cerebellar organotypic slices demyelinated with lysolecithin for 15-17 h showed very active remyelination following termination of the demyelinating treatment.

At 7 DIV, cerebellar OSCs were treated for 15-17 h with the demyelinating lipid lysolecithin, and then the toxic medium was switched back to the normal control medium, after which the slices were fixed either 2 days (10 DIV) or 13 days (21 DIV) later (A). A separate analysis at 10 DIV showed profound demyelination following treatment with lysolecithin, with a significant 43% decrease from the control values in the percentage of neurites covered by MBP in these slices (the statistical outcome is not shown in (B), in which the data collected at 10 and 21 DIV were grouped in one graph). At 21 DIV, following removal of lysolecithin and 13 days of incubation in the control medium for recovery, the number of neurites covered by MBP was now similar in this group to that in the control group (B), showing that a very active remyelination process had taken place during the time of recovery. Data are plotted as Mean ± SEM of N = 10 slices/group. Scale bar = 20 µm.

Culture type	Experiment	Termination of experiment (DIV)	Number of biological repeats	*N* number	Origin of individual datum	Independent variable 1	Independent variable 2	Dependent variable	Data sets passed normality test?	Statistical test	Post-hoc test	Corresponding figures
Rat glial precursor cells		4 or 7	3	3 biological repeats	Mean of 3 wells for each biological repeat	Treatment 0.2; 1; 5; 10 mM Phe	DIV 4 or 7	Total number of GalC-positive cells	N/A	Two-way ANOVA	Šídák's	Fig. S1
Oligodendrocyte-enriched		14	2	10 image locations	Mean of 3 cover glasses for each image location	Treatment 0.4; 1.2; 2.4; 4.8 mM Phe	N/A	Total number of MBP-positive cells	No	Kruskal-Wallis test	Dunn's	Fig. S2B
								Area covered by MBP-positive cells with membranous sheaths	Yes	One-way ANOVA	Dunnett's	Fig. S2C
Cerebellar organotypic slices	1	21	2 to 3	10 to 11 slices	Mean of 20 fields of view for each slice	Treatment 0.1; 0.6; 1.2; 2.4 mM Phe	N/A	MBP staining area	Yes	One-way ANOVA	Dunnett's	Fig. 2A
	2	4, 10, or 21				Treatment 2.4 mM Phe		MBP or Calbindin staining area; % of neurites covered by MBP	Yes	Unpaired, two-tailed t-test	N/A	Fig. S3 and Fig. 2B to 2D
	3	28				Treatment 2.4 mM Phe stopped at 21DIV; 2.4 mM Phe for 28 DIV		MBP or Calbindin staining area; % of neurites covered by MBP	No	Kruskal-Wallis test	Dunn's	Fig. 3A and 3B
									Yes	One-way ANOVA	Dunnett's	Fig. 3C
		10 or 21		5 to 7 slices		Treatment 0.5 mg/ml lysolecithin at 7 DIV for 15-17 h	DIV 10 or 21	% of neurites covered by MBP	N/A	Two-way ANOVA	N/A	Fig. S4
	4	10 or 21		10 to 11 slices		Treatment 2.4 mM Phe	DIV 10 or 21	Number of Iba1-positive cells; cell size of Iba1-positive cells	N/A	Two-way ANOVA	Šídák's	Fig. 4A and 4B

Table S1. Summary of the statistical tests performed in every experiment.

Three *in vitro* models of increasing complexity (rat glial precursor cell culture; oligodendrocyte-enriched cultures; cerebellar organotypic slice culture –OSCs) were used to test the effects of high Phe concentrations on myelination, in six experiments, out of which four were conducted with OSCs. The main experimental conditions of each experiment are reminded, and the statistical tests and variables used for each test are indicated, along with the figures to which they correspond.
